# Amyotrophic Lateral Sclerosis—The Complex Phenotype—From an Epidemiological Perspective: A Focus on Extrapyramidal and Non-Motor Features

**DOI:** 10.3390/biomedicines10102537

**Published:** 2022-10-11

**Authors:** Daniele Urso, Stefano Zoccolella, Valentina Gnoni, Giancarlo Logroscino

**Affiliations:** 1 Center for Neurodegenerative Diseases and the Aging Brain, Department of Clinical Research in Neurology, University of Bari ‘Aldo Moro’, “Pia Fondazione Cardinale G. Panico”, 73039 Tricase, Italy; 2 Department of Neurosciences, Institute of Psychiatry, Psychology and Neuroscience, King’s College London, De Crespigny Park, London SE5 8AF, UK; 3 Department of Basic Medicine, Neuroscience, and Sense Organs, University of Bari ‘Aldo Moro’, 70121 Bari, Italy

**Keywords:** amyotrophic lateral sclerosis, ALS, population-based, non-motor, parkinsonism, epidemiology

## Abstract

Amyotrophic lateral sclerosis (ALS) is the most common motor neuron disease (MND) and has emerged, among the disorders, with the largest increase in incidence in Western countries. Although the typical clinical phenotype of ALS involves simultaneous upper and lower motor neurons, there is growing evidence that the neurodegeneration during the course of the disease can also involve other motor and non-motor regions. In this review, we analyzed and discussed available data from epidemiological population-based studies on extrapyramidal and non-motor features during the course of ALS.

## 1. Introduction

Amyotrophic lateral sclerosis (ALS) is the most common motor neuron disease (MND), characterized by progressive degeneration of motor neurons in the cortex, the brainstem, and the spinal cord [1]. According to recent global burden of disease studies, MND has emerged in the last decade as the fourth most common cause of death among neurological disorders in the U.S.A., and among the diseases with the largest increase in absolute numbers in Western countries [2]. The typical clinical phenotype of ALS involves simultaneous upper (UMN) and lower motor neurons (LMN) and is usually fatal within 4 years of onset. Muscle weakness begins in a discrete body region (usually the face, arm or leg) and advances steadily over time and space. Less typical forms of the disease are characterized by much longer survival, or pure UMN or LMN involvement [1]. Clinical phenotypes of ALS can also involve other motor and non-motor regions. The most frequent is the presence of cognitive and/or behavioral impairment, which can be observed in up to 50% of patients; and in about 15%, it leads to the fulfilment of the diagnostic criteria for frontotemporal dementia (FTD) [1,3]. In addition to cognitive dysfunction, other systems can be involved, including extrapyramidal motor systems, sleep, and the autonomic nervous system [4]. Finally, other regions such as the hypothalamus may be implicated as defects in energy metabolism including weight loss, hypermetabolism, and hyperlipidemia have been associated with ALS [5]. Based on these observations, ALS can no longer be considered a disease limited to the motor system but rather a multisystem neurodegenerative disorder that involves other motor and non-motor domains [6]. This review aims to describe the available data from epidemiological studies on extrapyramidal and non-motor features during the course of ALS.

## 2. Extrapyramidal Motor Features

Table 1 includes population-based studies exploring the prevalence and association of extra-pyramidal and non-motor features in patients with ALS. The concurrence of frank levodopa-responsive parkinsonism and ALS has been observed endemically in Guam and the Kii Peninsula, and has been named Guam ALS/Parkinsonism Dementia Complex [7]. Furthermore, the presence of clinical ALS and Parkinson’s disease (PD), outside the known Guam and Kii Peninsula foci, has been described as a distinct and rare pathological entity, named Brait–Fahn–Schwartz disease, in which basal ganglia, corticospinal and anterior horn cell degeneration are merely part of a more encompassing disease [8]. However, mild parkinsonian features have been reported in patients with ALS [9]. Several studies have shown imaging [9,10] and pathologic [11,12] evidence of degeneration of substantia nigra and striatum in ALS.

The EURALS population-based study, which investigated the presence of parkinsonism features in 146 patients with newly diagnosed ALS, showed that patients with ALS have a higher than expected chance to report parkinsonian features [13]. The odds are almost five-fold for bradykinesia and six-fold for rigidity. In another prospective population-based study, about one-third of patients with ALS showed parkinsonism features meeting the diagnostic criteria for parkinsonism [14]. In the majority of cases, parkinsonism features were detected at the time of ALS diagnosis or during the follow-up, but also in the advanced stages. Patients with ALS and parkinsonism were more frequently male, but they did not show any other clinical or demographic difference compared with patients with ALS without parkinsonism signs. The neuropsychological profile did not differ between ALS with and without parkinsonism. Interestingly, a 123I-FP-CIT SPECT scan demonstrated that the dopaminergic nigrostriatal pathway was preserved, indicating that parkinsonian features in patients with ALS are likely to be related to extra-striatal brain pathways dysfunction [14]. In the same study, 18F-[18F]Fluorodeoxyglucose (FDG)-PET and MRI scans revealed that patients with ALS and parkinsonism have relative hypermetabolism in frontal regions, hypometabolism in the cerebellum, reduced cortical thickness in the left pre-central region, increased FA in the retrolenticular part of the internal capsule, and reduced FA in the sagittal stratum. These neuroimaging findings supported the hypothesis that ALS patients with parkinsonism have altered neuronal functions in extra-striatal brain regions involved in motion.

## 3. Non-Motor Features

A growing body of evidence from recent years suggests that ALS results in a wide range of non-motor symptoms, which can have a significant impact on patients’ quality of life [4]. The presence of non-motor symptoms in ALS is the clinical demonstration that ALS is a multisystem disorder. As for other neurodegenerative diseases, non-motor symptoms in ALS can arise from pathological disease spread of motor areas into neighboring non-motor regions of the brain [4]. However, unlike PD [15], non-motor symptoms in ALS have been poorly defined and their biological substrates have been scarcely investigated so far. Non-motor symptoms are not strictly correlated to an underlying motor pathology and can be largely categorized into neuropsychiatric, gastrointestinal, and autonomic disturbances. [4]. Among the non-motor features, depression, pain, weight changes, fatigue and cognitive impairment are common in ALS and can contribute to diminished health outcomes and reduced quality of life [16]. Remarkably, due to a lack of awareness by both clinicians and patients, some of the non-motor symptoms result to be underreported and underdiagnosed [15]. Although several case–control studies have investigated non-motor symptoms in ALS, there have been few population-based clinical studies of the prevalence of non-motor symptoms [4] (Table 2).

Depression is one of the major cognitive and psychiatric disorders reported by people with ALS, affecting between 6% and 29% of patients [4]. Roos and colleagues examined the relative risk of depression among patients with ALS, before and after diagnosis [17], in a nested case–control study based on Swedish national health and population registers. They found that patients with ALS had a highly increased risk of depression, even before diagnosis. Antidepressant use was more common in patients with ALS than in controls, especially during the year before and the year after diagnosis. Similarly, a population study in Sweden found a near sixfold increased risk for suicide among ALS patients over a 40-year period. The highest relative risk for suicide was observed within the first year after the patient’s first period of hospitalization [18]. Population-based studies have also long corroborated the relationship between psychotic events and ALS. This close association may underlie the prodromal nature of the extra-motor symptoms in the framework of ALS pathogenesis. It has been demonstrated, in fact, an increased risk of hospitalization due to schizophrenic symptoms in the 5 years preceding ALS diagnosis [19,20]. The risk of hospitalization due to a psychiatric disorder is higher 1 year prior to the onset of motor symptoms [19,20]. Furthermore, a register-based nested case–control study showed that family members of ALS patients, especially children, had an increased risk for manifesting psychiatric disturbances both before and after their relative’s diagnosis [20]. Similarly, aggregation studies suggested neuropsychiatric illnesses and ALS clusters in families. Two population-based cohort studies showed that the relative risk of developing a neuropsychiatric condition, such as schizophrenia, psychosis, obsessive-compulsive disorder, autism, and alcoholism, was significantly higher in first- or second-degree relatives of ALS patients [21,22]. Whether this can be explained by a shared polygenic risk between psychiatric diseases and ALS or by genetic pleiotropy of a few variants into several kindreds spectrum remains to be elucidated. Weight loss is another non-motor feature of ALS and has been investigated in a population-based setting by Marin and colleagues [23]. At the time of diagnosis, 50.6% of ALS patients reported a weight loss of more than 5%, and 36.0% of patients reported a weight loss of more than 10%. Furthermore, weight loss was independently associated with survival. Several observational studies have demonstrated that sleep disorders are frequent in ALS [24,25]. A recent systematic review showed that between 50–63% of patients with ALS reported poor sleep quality which was assessed by the Pittsburgh Sleep Quality Index (PSQI) [24]. This review also highlighted that poor sleep quality was associated with ALSFRS-R score, anxiety and depression but was not related to the type of ALS at disease onset and was not always associated with duration of ALS symptoms. To date, population-based studies of the prevalence of sleep problems in ALS are unavailable. Such studies are required to shed light on our understanding of sleep disorders in ALS. Chiò and colleagues investigated the prevalence and characteristics of pain in an epidemiological series of patients with ALS compared with a population-based control [26]. Patients with ALS reported pain more frequently than controls (56.9% vs. 33.1%), with pain frequency and intensity correlated with a worse functional score and a longer disease duration [26]. Pain in patients with ALS was more frequently located at the extremities and interfered with all areas of daily function, but patients reported a greater interference than controls in the domains of enjoyment of life and relation with other people. Patients with ALS were also more frequently prescribed non-opioid analgesics and opioids than controls. These population-based studies indicated that pain is an under-recognized and undertreated non-motor symptom in ALS. Population-based studies are lacking regarding other non-motor features in ALS. Further research is needed to characterize the prevalence of these features since they can worsen the prognosis and have a significant impact on the quality of life of patients and caregivers.

**Table 2:** Population-based studies of non-motor features in amyotrophic lateral sclerosis.

Author (Year)	Domain	Study Design, Country, *n*	Mean Age, Years	Findings
Fang et al., (2008) [18]	Suicide	Population-based study, Sweden, *n* = 6642 ALS	67.6	6-fold increased risk for suicide among ALS patients (SMR 5.8, 95% confidence interval 3.6–8.8)
Turner et al., (2016) [19]	Psychiatric disorders	Record-linkage study, U.K., *n* = NA	NA	Increased risk of hospitalization for schizophrenia in the year preceding ALS (rate ratio 2.95, 95% confidence interval 2.13–4.00). Increased risk of hospitalization for depression in the 5-year preceding ALS (rate ratio 1.50, 95% confidence interval 1.24–1.81).
Longinetti et al., (2017) [20]	Psychiatric disorders	Register-based nested case–control study, Sweden, *n* = 3648 ALS and *n* = 36,480 healthy controls	NA	Individuals with previous neurodegenerative or psychiatric diseases had a 49% increased risk of ALS (odds ratio 1.49, 95% confidence interval 1.35–1.66) Patients with ALS had increased risks of other neurodegenerative or psychiatric diseases after diagnosis (hazard ratio 2.90, 95% confidence interval 2.46–3.43)
Marin et al., (2016) [23]	Weight loss	Population-based study, France, *n* = 322 ALS	69.6	Weight loss > 5%: 50.6% of ALS patients Weight loss > 10%: 36% of ALS patients
Chiò et al., (2012) [26]	Pain	Population-based cross-sectional study, Italy, *n* = 160 ALS and *n* = 160 healthy controls	62.4	Patients with ALS reported pain more frequently than controls (56.9% vs. 33.1%)

## 4. Cognitive Impairment and the Frontotemporal Dementia

Traditionally, ALS has always been considered a disastrous disease that afflicts motor neurons and leads to devastating immobility whilst cognition remains intact [27]. This assumption has been debunked in recent years with numerous studies that have demonstrated the presence of cognitive and behavioral dysfunctions in ALS [6,28,29,30,31,32,33,34,35]. In particular, an association between ALS and FTD has been increasingly recognized, and with the advent of new genetic discoveries [29,30], as well as new neuropathological and neuroimages evidence, the concept of the frontotemporal dementia–motor neuron disease continuum has progressively emerged. Five population-based studies have explored the presence of cognitive impairment and frontotemporal dementia–motor neurons (Table 3) [6,31,32,33,34]. Evidence has shown that patients with ALS may present executive dysfunction, personality changes, poor insight, obsessions, aggressiveness, irritability and verbal fluency alteration.

Up to 40% of patients with ALS present with cognitive impairment, whilst between 7–20% of patients with ALS also fulfilled the criteria for frontotemporal dementia at baseline [6,31,32,33,34]. Specifically, the ASL–FTD spectrum is consistent with features of behavioral variant FTD. The main function found to be predominantly affected is the executive function which resulted as being impaired in 20–30% of patients with ALS. Non-executive cognitive impairment is found in around 2–10% of patients [31,32,33], whilst 6–13% of patients showed isolated behavioral impairment. However, depending on the cognitive tests and tools used in the different studies, 50% of ALS patients may not have any cognitive deficits [31,32,33]. Interestingly, decline in cognitive function was faster in patients who were cognitively impaired at baseline, and normal cognition at baseline was associated with a tendency to remain cognitively intact, and with slower motor and cognitive progression [33]. Further longitudinal population-based studies are needed to define the patterns of cognitive progression in patients with ALS.

A recent population-based cross-sectional study assessed the association of the degree of severity of motor impairment to that of cognitive impairment in a large cohort (n = 797) of patients with ALS [31]. According to King staging, the frequency of cases with ALS–FTD progressively increased from 16.5% in stage 1, to 44.4% in stage 4. On the other hand, the frequency of ALS patients with cognitive and/or behavioral impairment (without dementia) increased from King stage 1 to King stage 3 and decreased thereafter, suggesting that intermediate cognitive categories may represent a transitional condition between normal cognition and FTD. Furthermore, ALS–FTD was more frequent in patients with bulbar involvement [31]. These findings indicate that ALS motor and cognitive components may worsen in parallel, and that cognitive impairment becomes more pronounced when bulbar function is involved. Previous population-based approaches have also shown that cognitive dysfunction, especially executive impairment, is associated with worse motor prognosis in ALS [32,33] and that patients with ALS–FTD are typically older, have a lower education level and shorter survival compared with non-demented ALS [32]. The association between bulbar involvement and dementia emphasized in these population-based studies [31,32] support the hypothesis that ALS pathology disseminates in a regional ordered sequence, through a cortico-efferent spreading model. A recent population-based study explored the impact of executive dysfunction on social cognitive performance in patients with ALS [35]. The authors found that executive dysfunction mediates performance on social cognition. On the other hand, the development of motor neuron disease in patients who initially presented with FTD has been scarcely studied. Interestingly, a clinical and neurophysiological study on 40 patients with FTD, found that 12.5% of cases had concomitant motor neuron disease and a further 27.3% of the patients with FTD had clinical evidence of minor motor system dysfunction such as occasional fasciculations, mild wasting or weakness [36].

A recent population-based study found that FTD at diagnosis was present in 10% of patients with ALS who underwent genetic analysis; the vast majority of cases (> 70%) did not carry genetic mutations, whereas 23% had C9orf72 expansion and 2% FUS mutation. There were no patients with SOD1 gene mutation among those presenting with FTD at diagnosis [6]. Patients with C9orf72 expansion had a 6-fold higher risk of developing ALS and FTD, compared with patients without mutations. Data from a population-based register of patients with ALS in Ireland showed that 50% of patients with C9orf72 repeat expansion showed co-morbid FTD, compared with 12% of patients without the expansion [37].

## 5. Conclusions

Our review of available population-based studies confirms a growing body of evidence that ALS involves a wide range of motor and non-motor symptoms, which can have a significant impact on patients’ quality of life. Some of the non-motor symptoms may represent the direct consequence of motor dysfunction (e.g., pain and immobility), and some are not related to the motor dysfunction (e.g., cognitive and behavioral impairment) and may be localized neuroanatomically elsewhere. Better recognition of these symptoms may help improve our understanding of the etiopathogenesis of ALS and support the concept of ALS as a multisystem disorder.

Data from population-based studies indicated that about 7–20% of patients with motor neuron disease met the criteria for a diagnosis of frontotemporal dementia (ALS-FTD) at baseline, and cognitive impairment without dementia might be detected in a higher proportion of patients. Most of the studies have indeed observed that one-half of patients do not have cognitive abnormalities. Moreover, cognitive dysfunctions progressively increase consistently with the severity and diffusion of motor impairment, indicating that motor and cognitive features may worsen in parallel, and that cognitive impairment becomes more prominent when bulbar function is involved. Preliminary data seems to indicate that patients with genetic ALS due to C9orf72 expansion are at higher risk of developing the spectrum ALS–FTD, although these data need further confirmation. Among the other non-motor symptoms, depression is the major manifestation encountered by people with ALSs. Depression can occur even before the diagnosis of MND. Data from population-based studies also indicated that pain is an under-recognized and undertreated non-motor symptom in ALS.

Our analysis of available data revealed that the extra-pyramidal involvement is not uncommon during the course of ALS, even at the early stages of the disease. Population-based studies demonstrated that the prevalence of parkinsonism in patients with ALS is about one-third, and that patients with ALS at the time of the diagnosis have a 5 to 7-fold higher risk of showing parkinsonian features. The pathogenesis of extrapyramidal involvement in ALS is different from Parkinson’s disease and neurodegenerative parkinsonism as the dopaminergic nigrostriatal pathway seems preserved.

People with ALS experiencing greater frequency of non-motor symptoms report lower quality of life than those who indicate more severe motor symptoms, suggesting that the impact of these non-motor symptoms on the daily lives of people with ALS is at least comparable to the impact of motor symptoms [38,39]. However, non-motor symptoms have not been consistently evaluated in trials for people with ALS, and when evaluated, non-motor symptoms were primarily assessed using instruments and impairment cut-offs that are not adapted for people with ALS [40].

Future population-based studies should focus on evaluating the prevalence and characteristics of other non-motor symptoms, such as sleep disturbances, in people with ALS. Epidemiological research is essential to provide new data on non-motor and extrapyramidal features in ALS and to improve policy-making in public health intervention [41,42].

## Figures and Tables

**Table 1:** Population-based studies of parkinsonian features in amyotrophic lateral sclerosis.

Author (Year)	Domain	Study Design, Country, *n*	Mean Age, Years	Prevalence
Pupillo et al., (2015) [13]	Parkinsonism	Population-based cross-sectional study, Italy, *n* = 146 ALS and *n* = 146 healthy controls	NA	Rigidity: 8.2% of cases vs. 2.1% of controls Bradykinesia and postural instability: 8.2% of cases vs. 2.7% of controls
Calvo et al., (2019) [14]	Parkinsonism	Population-based prospective study, Italy, *n* = 101 ALS	65.1	Parkinsonism: 30.7% of cases

**Table 3:** Population-based studies of cognitive features in amyotrophic lateral sclerosis.

Author (Year)	Domain	Study Design, Country, *n*	Mean Age, Years	Findings
Phukan et al., 2011 [34]	Cognitive impairment	Population-based cross-sectional study, Ireland, *n* = 160 ALS and *n* = 110 healthy controls	63.5	13.8% of ALS patients had FTD 34.1% of ALS patients had cognitive impairment (executive) 14% of ALS patients had nonexecutive cognitive impairment 46.9.1% of ALS patients had no cognitive impairment
Montuschi et al., 2013 [32]	Cognitive impairment	Population-based cross-sectional study, Italy, *n* = 183 ALS and *n* = 127 healthy controls	67	12.6% of ALS patients had FTD 19.7% of ALS patients had cognitive impairment (executive) 5.5% of ALS patients had non-executive cognitive impairment 6% of ALS patients had behavioral impairment 6% of ALS patients had non-classifiable cognitive impairment 49.7% of ALS patients had no cognitive impairment
Elamin et al., 2013 [33]	Cognitive impairment	Population-based longitudinal study, Ireland, *n* = 186 ALS	63.4	11.8% of ALS patients had FTD 25.2% of ALS patients had cognitive impairment (executive) 12.3% of ALS patients had non-executive cognitive impairment 50.5% of ALS patients had no cognitive impairment
Chiò et al., (2019) [31]	Cognitive impairment	Population-based cross-sectional study, Italy, *n* = 797 ALS	65.5	20.5% of ALS patients had FTD 4.8% of ALS patients had cognitive and behavioral impairment 16.6% of ALS patients had cognitive impairment 2.0% of ALS patients had non-executive cognitive impairment 7.9% of ALS patients had behavioral impairment 48.2% of ALS patients had no cognitive impairment
Gianferrari et al., (2022) [6]	Cognitive impairment	Population-based cross-sectional study, Italy, *n* = 1613 ALS	67.01	7.01% of ALS patients had FTD
Burke et al., (2016) [35]	Social cognition	Population-based case–control study, Ireland, *n* = 106 ALS and *n* = 50 healthy controls	60.4	Executive dysfunction impacts on social cognitive performance

## Data Availability

Data are contained within the article.
